# Dr. Lubbock, on Diabetes

**Published:** 1801-01

**Authors:** Richd. Lubbock

**Affiliations:** Norwich


### *To the Editors of the Medical and Phyſical Journal.*

GENTLEMEN,

ENCLOSED you will find ſome remarks upon the nature and treatment of Diabetes; they are the reſult of an impartial and attentive inquiry; and if you think proper, I wiſh them to be inſerted in the Medical and Phyſical Journal.

I am,

GENTLEMEN,

*Norwich, Dec.* 8, 1800. Your obedient ſervant,

RICHD. LUBBOCK.

---

In the hiſtory and treatment of the morbid actions of the living body, experience tells us there is much difficulty in tracing any given effect to its appropriate cauſe; and the ſource of this difficulty will, by the reflecting mind, be readily and truly referred to the intricate ſtructure of the human machine, acted upon by a variety of cauſes, whoſe influence from their nature can be but remotely inferred, and which muſt of courſe frequently either eſcape or delude the eye of the niceſt obſerver.

I have been led to this train of thinking, from the too general diſappointment which medical characters ſuffer, in reducing to practice modes of treatment, in the opinions of their authors and inventors, ſaid to be effectual and certain in the removal of certain diſeaſes.—Many years have not elapſed, ſince the medical world was cheered with the proſpect of relieving cancerous affections by the aid of cicuta; and for ſome time we have been led to ſuppoſe mercury an antidote to hydrocephalus.—To enumerate ſimilar or more recent inſtances will be unneceſſary; it will be ſufficient to remark, that within the laſt two or three years I have experienced a ſimilar diſappointment, in my attempts to relieve ſeveral diabetic patients who have fallen to my care; and from what I have ſeen of this diſeaſe, I am led to think, that its mode of cure ſtill remains to be diſcovered.

In the full and ingenious publication of Dr. Rollo, a mode of treatment, ſaid to be efficient in diabetes, has been given to the medical world; but I think, in the ſubſequent obſervations, it will appear, that this mode has proved unſucceſsful in many caſes, although its application has been conducted with candor, and carried on with perſeverance.

I ſhall proceed therefore to detail the hiſtory of ſome caſes of this diſeaſe, which were taken into the Norfolk and Norwich hoſpitals, the event of which, it would ſeem, has been unfavourable vourable to Dr. Rollo's theory and treatment of it. And from the obfervation which I have been able to make upon this difeafe, I think I am warranted in faying, that a leaning towards what is marvellous has pervaded, and in fome meafure diftorted, the hiftory of its fymptoms and phenomena from the earlieft times.

Thomas Smith, æt. 39, by trade a fhoemaker, was admitted into the hofpital, Dec. 21, 1799; he complained of an emaciation, which, he faid, had commenced about two years, and which had increafed more particularly during the laft twelve months. From his fhrinking, the outlines of the face had become fo ftrong and prominent, as to convey to the moft common obferver, a ftate of great difeafe. His fkin was harfh and dry, and apparently altogether impervious to the perfpirable fluids; he complained of great thirft, and of a fenfation of heat about the ftomach; and upon inquiry, faid he made a large quantity of urine. His appetite was good, and the abdomen was fomewhat enlarged, but there was no particular irregularity of bowels. He had no affection of the prepuce, but complained of weaknefs of the loins; his fight was alfo weak, and the verge of the eye-lids was red and flightly inflamed, and the gums were fpongy and ulcerated. He had no particular pains. From the emaciation, the direction of the mufcles and the courfe of the veins over the whole furface of the body were fo diftinct as to denote the total abfence of fatty matter. But notwithftanding this appearance of difeafe, he poffeffed confiderable mufcular power, (a circumftance peculiar almoft to diabetic macies) and had been able to work at his bufinefs. His mode of living had not been marked by great intemperance, and he had only lately, from thirft, indulged in a freer ufe of malt liquor and other liquids. Having feen him a week previous to his admiffion into the hofpital, I defired him, during that interval, at different times, to meafure the quantity of urine paffed in twenty-four hours, and he found it vary from twenty-nine to thirty-one pints. After his admiffion, his urine was regularly collected, and meafured in the prefence of the houfe apothecary, every twenty-four hours, beginning from Sunday noon, Dec. 22. The following table will exhibit the quantity of urine paffed daily, for the firft few days; but a daily report being tedious and unneceffary, afterwards the greateft and fmalleft quantities paffed during longer intervals are only noted. The figures above the line denote the dates; below the line, the quantity of urine paffed. There will alfo be found fubjoined, tables of the urine paffed by two other diabetic patients. Of Richard Fofter, æt. 37, by trade a carpenter, who was admitted into the hofpital, January 26, 1800, and who previoufly paffed about 12 pints of urine daily;—of James Brown, æt. 43, by trade a weaver, admitted

April 6, 1799, who paffed from ten to fourteen pints daily, previous to admiffion.

### SMITH.

| Urine paffed in 24 hours previous to admiffion. | Dec. 22 to 23 | 24 | 25 | 26 | 27 | 28 to 31 | Jan. 1 to 31 | Feb. 1 to 28 | Mar. 1 to 24 |
|---|---|---|---|---|---|---|---|---|---|
| Pints 29 to 31 | 18 | 13 ½ | 9½ | 8 | 6 | 5 | 4 ¾ to 4 | 4 ½ to 4 | 4 ½ to 4 |

### FOSTER.

| Urine paffed in 24 hours previous to admiffion. | Jan. 26 to 27 | 28 | 29 | 30 | 31 | Feb. 1 to 28 | Mar. 1 to 23 |
|---|---|---|---|---|---|---|---|
| Pints 12 | 9 ½ | 8½ | 8 | 8 | 7 | 6 to 4 ½ | 5 to 3 ½ |

### BROWNE.

| Urine paffed in 24 hours previous to admiffion. | April 6 to 7 | 8 | 9 | 10 | 11 | 12 to 30 | May 1 to 26 |
|---|---|---|---|---|---|---|---|
| Pints 10 to 14 | 6 | 5¼ | 4¼ | 5 | 4¼ | 3¾ to 1 ½ | 3 to 2 |

Of thefe patients, two were of what is termed the fanguine temperament, having fair complexions and light hair; the third was fomewhat florid, but had dark hair. I have mentioned the particulars of Smith's fymptoms, his cafe being the more ftrongly marked; and as the fymptoms of the other two cafes differed from his only in degree, excepting that Browne's prepuce was affected with phymofis, and Fofter's gums were not affected, I was of opinion any detail would be ufelefs. It fhould be obferved, that the urine of all thefe patients was whey coloured, fweet to the tafte, and had a violet fmell.

The fame plan of diet was purfued by all thefe patients; they were all allowed four pints of liquid daily, confifting of two pints of milk, and of two pints of meat decoction, for the moft part beef, without any oatmeal or vegetable matter, and they had meat twice daily, viz. for dinner, and a fmaller portion for fupper. Bread and beer and every article of vegetable preparation were prohibited, and I have the greateft reafon to fuppofe thefe regulations were ftrictly adhered to. It muft be obferved, the milk and meat decoction were meafured with a common tin beer meafure, and the meafure by which the urine difcharged was eftimated was apothecary's meafure; and upon a comparifon I found the pint beer meafure ufed, contained an ounce and a half more than the apothecary's pint, fo that the four pints of liquid daily taken, would exceed in quantity four apothecary's pints of urine difcharged by fix ounces, or be equal to four pints fix fix ounces; and it muft alfo be recollected, in addition to the four pints taken, that thefe patients daily took from fix to eight ounces of water, as a vehicle to the medicines which were thought neceffary, fo that the fum of the liquid daily taken may be faid to be nearly equal to five pints, apothecary's meafure.

The medicines taken were kali præparatum, natron præpar. aqua ammoniæ pura, kali fulphurat, ammonia hepatizat. ferrum ammoniacale, &c. and they were given in full dofes, and for the moft part under the infpection of the apothecary.

This detail being premifed, I fhall proceed to offer a few remarks, naturally arifing during the treatment of thefe cafes, which, if well founded, may, by an application to Diabetes in general, afford us more precife and accurate notions of it.

The circumftance more immediately and ftrongly affailing the attention, after the admiffion of thefe patients into the hofpital, was the immediate and fudden reduction of the urine, which, as may be feen from the annexed tables, took place in a very few days, and in a degree fo as not to exceed the quantity of liquid taken. This change taking place fo rapidly, and before the quality of the diet ordered, or the effects of the medicines prefcribed, could have influenced the fyftem, awakened in my mind no fmall doubt of the truth of a propofition concerning this difeafe, which has been univerfally admitted as true, from the time of Aretæus to the prefent day; viz. that in this difeafe, the difcharge of urine, in any given time, exceeds the liquid taken in the fame time. The facts arifing from the cafes above ftated, tend in my mind to weaken the truth of this propofition, by fhewing, that the quantity of urine paffed bears a more direct ratio to the quantity of liquid taken, and by no means exceeds it. That this is the cafe, I infer from the fore-mentioned tables; and from our ignorance of any procefs or law of the animal œconomy, by which the liquid taken can, as fuch, be augmented in quantity; and laftly, from the various modes of treatment and theories of this difeafe, which have been foftered by the imagination, with the fole view of preventing and explaining this multiplying procefs.

On this fubject phyfiology affords us no affiftance, and accurate obfervation tells us, that the bafis of all the liquids either remaining in, or difcharged from, the animal machine, muft have been received from without; and an attention to the foregoing tables will fhew, that after the firft few days, the urine daily difcharged, continued, for weeks and months, to be lefs than the liquids taken.

From thefe facts and reflections, I am induced to deny that part of the hiftory of this difeafe, that fays the quantity of urine paffed in a given time, furpaffes the liquid taken.

 And

And in carrying our inquiries farther, and to the origin of this fuppofition, it is rendered probable that it commenced in the obfervations, either made by Aretæus himfelf, or received by him from others, he having exprefsly and in terms faid, "that the quantity of liquid taken is not equal to the quantity of urine, for that there is more urine paffed." I am fully aware, that Aretæus is ever quoted as a model of precifion and accuracy in the detail of his hiftory of difeafes, and to doubt what antiquity has fanctioned, and habit has confirmed, may be thought a bold, perhaps an invidious undertaking; yet, I hope I fhall not be charged with want of candour, when I fay, from a review of the hiftory of Diabetes, as delivered by this author, that it is more than probable his wonted judgment has not been exercif-ed in the detail of its fymptoms, either from the little opportu-nity which he had of feeing it, or from feeing it with a mind be-numbed by the impulfe of aftonifhment. That fuch is the cafe, his firft remark will evince, in which he fays, "that a wonder-ful phenomenon is the affection of Diabetes, and very rarely happening to man." And afterwards, in explaining more parti-cularly the caufes of this difeafe, and confidering it "as a deli-quefcence or converfion of the folids into a liquid form," it would feem, that Aretæus found, to account for, and get rid of the folids fo converted into liquid, a fecond fuppofition was effentially neceffary, giving origin to the opinion in queftion, "that the liquid difcharged by urine exceeded the liquor in-gefta."—This feems to me the origin of an error in the hiftory of this difeafe; and to this fource the fanciful details of cafes, the ftrange perverted relations in the collections of Schenkius, as well as of others, may be truly traced.

If, however, it be proved, that the urine difcharged does not exceed in quantity the liquid taken, an important conclufion readily prefents itfelf to the mind, which is, that the emaciation taking place in this difeafe, does not arife from any lofs fuftained by an undue or increafed quantity of urine beyond the liquid taken; and therefore, fome other caufe is neceffary to the ex-plaining this phenomenon. On the fame ground it may be ob-ferved, that the modes of treatment, inftituted folely with a re-ference to the increafed quantity of urine, are erroneous; and as fuch, the rubbing the furface with oil or unctuous fubftances, to preclude abforption from the atmofphere, is unneceffary and inefficient, as refulting from a principle falfe and imaginary. Neither, to explain this furplus of urine, is the ingenious con-jecture of Dr. Rutherford better founded, conftituting the lungs an elaboratory of water, and giving them the function of form-ing water, by the union of the hydrogen of the blood with the oxygen of the atmofphere. And having rendered it improbable that that the emaciation of Diabetes is connected with the quantity of urine difcharged, I fhall proceed to confider the quality of Diabetic urine as marked by a faccharine or honied tafte. This quality, obferved firft by Willis, has attracted the attention of later authors, and more particularly of Dobfon and Rollo, who finding that a greater quantity of extractive matter is produced by evaporation, from a given quantity of diabetic than from the fame quantity of healthy urine, have confidered this faccharine product as a probable caufe of diabetic emaciation. But it may be doubted, and perhaps with juftice, whether the increafed portion of faccharine extract in diabetic urine, be a caufe adequate to the explanation of the ftate of fhrinking, and whether it be not rather an effect or coincidence than a caufe.— In determining this queftion, it will be neceffary,

1ft. To compare the extractive matter produced, by evaporating equal quantities of diabetic and of healthy urine.

2nd. To compare the quantity of extractive matter obtained from a given quantity of diabetic urine, with the extractive matter contained in the fame quantity of liquid taken. And,

Laftly, To determine whether the concurrence of any other caufes may not contribute to the increafe of extractive matter in diabetic urine, without fuppofing that the deftruction of the folids contributes to fuch increafe.

With a view to the determination of this queftion, a pint of Smith's urine was evaporated, at four different times, and the extractive matter was carefully collected; viz. a pint was evaporated immediately upon his admiffion into the hofpital, at the period when he was paffing about thirty pints of urine daily, and, as near as may be, an ounce of faccharine extract was produced. In about a week, or as foon as the urine was reduced to the limited quantity of liquid ordered, another pint was evaporated, yielding of extract fourteen drachms. In a month or five weeks after the adoption of his curative plan, a third pint gave only fix drachms of extract; and about a week before his difcharge, another pint gave about an ounce of extract.

To explain the fourteen drachms of extract found in evaporating the fecond pint, it may be obferved, that this experiment took place as foon as the urine paffed was reduced fo as not to exceed the quantity of liquid daily ordered, and therefore it was probable that all the extractive matter contained in the greater quantity of liquid taken, previous to his admiffion, when he indulged his thirft (not to notice the folid) was not yet eliminated from the fyftem; fo that the liquids taken, being abridged to a given and much fmaller quantity, there was found, for a fhort interval, in every pint of urine, a greater and unufual ufual proportion of extractive matter. This explanation being admitted, it will appear, that the extract of Smith's urine, accurately fpeaking, varied only from an ounce to fix drachms.

To find the difference between this quantity of extract and that found in healthy urine, five pints of urine taken from healthy perfons were cautioufly and feparately evaporated, and it was found the extract produced, varied from half an ounce to rather more than fix drachms for each pint. Hence then it would appear, that the difference of the extractive products of healthy and diabetic urine is not fo great as to afford a probable caufe of the emaciation in diabetes;—and this will appear more fully hereafter.

But, in fpeaking upon this fubject, permit me to fay, that a fingular concurrence of events, but remotely connected, has caught my attention in this difeafe; I refer to the conftant coincidence of the parched and unperfpiring fkin with the faccharine quality of the urine; this concurrence has induced me, at times, to believe that in this difeafe the want of perfpiration, or of the excretion from the furface, was connected with the faccharine quality of the urine; and this belief has received farther fupport from an examination of the nature of the perfpirable fluids, and of the component parts of faccharine matter. It has been proved that fugar is, for the moft part, compofed of carbon, oxygen, and hydrogen united in a certain ratio; and it appears, by the experiments of Cruikfhank and Abernethy, that, befides the occafional aqueous fluid difcharged daily from the furface of the body, about three gallons of carbonic acid are alfo loft to the fyftem by the perfpirable matter. Now fuppofing, as happens in diabetes, this perfpirable excretion, or carbonic acid, is fuppreffed and retained in the fyftem, it is probable that the carbon and oxygen of the acid fo retained, by entering into a due combination with fome portion of the hydrogen of the animal body, may tend to the production of the faccharine matter of the urine; and as the carbonic acid is the general product of the vegetable world, it would follow, that its retention, in the animal body, may produce the phenomena of the defective animalization, characterifing diabetes, as exhibited in the formation of fugar.

Thus, in the preceding remarks, have I attempted, and I truft with impartiality, to invalidate certain data concerning the hiftory and theory of diabetes; I have attempted to difprove the generally received notion, that in diabetes the urine is difcharged in a greater ratio than the liquid taken; and I alfo truft I have rendered it improbable that the quality or quantity of the extractive matter difcharged by urine, is a principal caufe of the emaciation; having fhewn that fuch extract may more juftly juftly be referred to that contained in the liquid and folids taken into the body; and having made it probable that its faccharine quality may depend upon, or be connected with, the fuppreffed perfpirable matter. Hence then it is evident, that any mode of cure, or plan of treatment, inftituted folely with a reference to the increafed quantity, or altered quality of the urine, muft be defective and unfuccefsful; and, therefore, proceeding upon thefe erroneous principles, it will ceafe to be a matter of furprize, that, in the above cafes, I fhould have adopted the fuggeftions of Dr. Rollo, without the advantages I had been taught to expect. In the three cafes given above, a diet confifting folely of animal food, with the free and full ufe of the different alkaline medicines, including a liberal ufe of hepatized ammonia, effected little or no change in the general health. —And in Smith's cafe, although thefe regulations of diet and medicine were rigoroufly attended to, I cannot fay that any great or obfervable change was induced in the quality of the urine, which feemed, in a great meafure, to retain its faccharine tafte and violet fmell. And in this cafe, fuppofing that the milk taken might keep up thefe appearances, inftead of the two pints of milk two additional pints of ftrong meat decoction were fubftituted, fo that nothing but highly animalized food was taken; and under this plan he remained fome weeks, but without any real advantage. At length, after a due perfeverance, and becoming tired of the little variation of his food, his diet was ordered, on a fudden, and without any gradation, to be changed to the common hofpital diet, confifting of bread, vegetables, meat, malt liquor, and milk pottage, adhering however to the taking, in liquids, only four pints daily. By this alteration of the nature of the liquids and folids taken, no marked change in the quantity or quality of the urine was induced, and he remained in the hofpital fome time without any change in the urinary difcharge.

But it may be faid, that this patient had been in the ufe of animal food fo long, that his fyftem was rendered proof againft the influence of vegetable or mixed diet, as fhewn by increafing the quantity or the faccharine quality of the urine; — in anfwer to which, it muft be permitted me to remark, that Fofter, another patient, had been, at the time in which Smith's diet was changed, only about a fortnight in the hofpital, and had only for that time purfued the animal diet and alkaline medicines, and the urine difcharged was in the ratio of the four or five pints of the liquids taken, as the table will fhew; and to determine what change would be induced in the urine difcharged after fo limited an ufe of animal food and alkalies, he was alfo ordered, on a fudden, to take the ufual diet of the houfe, houfe, confifting of vegetables, bread, malt liquor, meat, &c. but, on a cautious inquiry, it did not appear that the urine was increafed in quantity; and in quality, fo far from exhibiting a more wheyifh or faccharine appearance, it was evidently, for fome days, of a higher or more amber colour.

After this ftatement, the power of a vegetable or mixed diet, in re-producing diabetes, by increafing the quantity, or altering the quality of the urine, may with reafon be doubted.

In the treatment of diabetes, patients are led, by the attention given by medical men to the quantity and quality of the urine, to fuppofe the fole caufe of their difeafe to be connected with the ftate of the urine; and, therefore, whenever either the quantity of urine is obferved to be reduced to the natural quantity, or the quality of the urine is deemed more favourable, they fall into the greateft delufion refpecting their general health, by afferting in terms that they are becoming well, or free from difeafe, although, at the fame time, the degree of emaciation is evidently growing worfe and advancing. This delufion was ftrongly exemplified in the three cafes before mentioned, their language being, from the decreafe of their urine, that they had loft their difeafe, although at the fame time they were gradually lofing flefh; and it is not to be doubted, but that, at no diftant period, they will fall victims to the emaciation. And to the fame end, it may be obferved, in the various cafes reported in the accurate publication of Dr. Rollo, that very few, if any, excepting Capt. Meredith, feem to have regained their flefh, although, from the urine being in quantity abridged by an abridgment of the liquids taken, they were deemed to be cured. This continuance of the emaciation may be remarked in the cafe of Clarke, fo accurately reported by Dr. Gerard; for this patient's urine being reduced, he was difcharged as cured by his phyfician, even at a time when his emaciation was ftationary, or even increafing; for being weighed upon the day of admiffion into the Liverpool Hofpital, and upon the day of his difcharge, it was found his weight had decreafed one pound. And the continuance of the macies in the cafes fo well defcribed by Dr. Cleghorn, and in the other cafes reported in S. Rollo's book, proves the difeafed ftate to have been unaltered, and to have refifted the curative means made ufe of. And I have no hefitation in faying, that it is probable, fince the appearance of Dr. Rollo's book, containing thofe cafes, many of them are fuffering from greater emaciation, or may have paid the debt of Nature: And it would be an ufeful document to lay before the public, could Dr. Rollo procure from his correfpondents the particulars of every cafe that he has printed, fhewing their fubfequent progrefs; by which it might might be known in what degree we may expect a fatal or falutary termination of this difeafe.

From all that has been faid, it would feem, that the leading circumftance in diabetes is the macies, which, from the experience which I have had of this difeafe, frequently comes on flowly and tacitly, the fubjects of it not perceiving or not feeling much deviation from health: In this way, I have known perfons for three or four years affected with diabetes, but poffeffing a degree of ftrength equal to the various exertions of pleafure or of bufinefs; and under this degree of diabetes, I have, at this time, two patients, who, excepting a flight and very gradual wafting of the body, fome increafe of thirft, with occafional uneafy fenfations about the ftomach, to which may be added, feeblenefs of fight at times, and an habitual tendency to phymofis, poffefs that degree of apparent health and ftrength that would miflead a common obferver. And to treat patients with fo flight a deviation from health, it muft be allowed, is an arduous aud difficult enterprize; plans of diet may be prefcribed, and medicines may be ordered, and the nature of their complaint may be duly pointed out to them, yet, irregularity in their application will neceffarily occur, and a frequent violation of all the neceffary falutary proceedings will take place; and, perhaps, from this caufe it has happened, that I have not found even this incipient ftate of macies in diabetes removed; and when it is more advanced, I have never feen it yield to any curative meafures, but it has continued to increafe more and more, till after a lingering and protracted wafting of the folids, the unhappy fufferer fometimes difplaying a countenance the moft hollow and hagard, with the different members, as it were, barely enveloped with the fkin, at other times prefenting an afpect bloated with anafarcous fwellings, finks gradually into the arms of death. And as has been before obferved, in this difeafe confiderable mufcular power remains with great emaciation; and this circumftance, I believe, has led very judicious practitioners to conclude, that diabetic patients have been materially relieved by certain curative means. But it is the emaciation that claims the firft attention of the phyfician, for, without the removal of this fymptom, neither the reduction of the urine, the alteration of its quality, nor the appearance of confiderable remaining mufcular power, will prove that the difeafe has yielded to any curative procefs.

Thefe obfervations may have been lengthened beyond the limits intended; fhould they, however, in any degree tend to convey to the mind, notions more accurate of the nature and treatment of diabetes, my principal object, the promotion of medical fcience, will be attained. And I fhall now only fubjoin a few general conclufions; and,

1ft. From what has been faid, it would feem, that in the nofological arrangement of diabetes, authors have materially erred, as it fhould neither be claffed with Cullen among the fpafmi, nor with Sauvage and others among the fluxus and profluvia; according to Cullen's arrangement, it fhould fall under the clafs cachexia, and in the order marcores; and were I afked to give a definition of it, I fhould give the following, keeping in mind the conjecture of the connection of the retention of perfpiration with the faccharine quality of the urine, as caufe and effect: I fhould define it,

Macies (anidrotica) cum cute non perfpirante, cum urina mellita, et plerumque multa ex multo potu, et haud raro cum palpebris, præputio et gingivis inflammatis.

And as from experience it would feem that this fpecies of macies has hitherto admitted of no fubftantial relief from medical treatment, I am difpofed to confider it with Selle and Neitzkius, as a difeafe of the lymphatic fyftem. It muft be underftood, that I am here fpeaking of what may be termed idiopathic diabetes, and not of that fpecies which Sydenham and others have mentioned as fupervening as a fymptom or fequela of acute difeafe, and which is of fhort duration.

2d. It is proved, that in diabetes the urine difcharged is in a direct ratio of the liquids taken; fo that in the cafes reported by Dr. Rollo, it would feem, as the macies remained unaltered, that all that was effected by the curative plan was, by abridging the quantity of liquid taken to abridge the urine difcharged.

3dly. And if the urine difcharged follows the liquid taken, it is evident the various alkaline preparations cannot influence the quantity of the urine; and from what has been faid, it may be much doubted, whether the quality of the urine is materially affected by them.

Laftly. The occurrence of phymofis has been mentioned by my friend, Dr. Girdleftone, as a conftant or pathognomonic fymptom of this difeafe; but I find this fymptom is dependent upon the formation of the prepuce, for in Smith and Fofter, the worft cafes which I have met with, this fymptom was abfent. This fymptom has, however, taken place in four other cafes which have fallen to my care, two of which poffeffed fo much apparent health, that the obftinacy of this fymptom afforded a clue to the unravelling of the nature of the complaint.

And in treating of the extractive matter of diabetes, authors feem to have confined their attention to the condition of the urine, urine, or of the liquid difcharged, confidering fuch matter as derivable folely from the fyftem, without carrying their thoughts to the examination of the extractive matter contained in the liquid received and taken into the body. Hence, if Smith paffed thirty pints of urine daily, each pint yielding an ounce of extract, it would feem as if they concluded, that thirty ounces of extractive matter were taken from the fyftem daily. But let it be remembered, that this man took daily thirty pints of liquid, confifting of porter and other malt liquor; of milk, water, &c.; that his appetite being good, he took freely of bread, meat, vegetables, &c.: Then fuch liquids and folids will readily afford a caufe adequate to the production of the thirty ounces of extractive matter contained in the thirty pints of urine paffed.

Hence it is probable, the quality of the urine is not the caufe of the fhrinking, for the origin of the extractive matter found in the urine may be referred to that taken in by the liquids and folids, without recurring to any lofs fuftained by the folid compages of the body and its confequent emaciation.

Farther, thefe arguments againft the faccharine matter as a caufe of the fhrinking in diabetes, will receive a confiderable acceffion of ftrength, from a comparifon of the nature and quantity of the liquids and folids taken daily by Smith after he had been in the hofpital fome time, with the quantity of extractive matter difcharged by urine daily. In this cafe, two pints of good milk, and two pints of ftrong meat decoction, were daily taken; and alfo for dinner, not lefs than from fix to eight ounces of boiled beef or mutton; and for fupper, not lefs than from three to four ounces of the fame meat were allowed. And after he had ufed this plan for fome time, it was found by experiment, he difcharged daily from four pints to four pints and a half of urine, each pint yielding only fix drachms of extract; fo that, in twenty-four hours, little more than three ounces of extractive matter were difcharged, a quantity which by no means equalled the extractive matter contained in the milk and the liquids taken daily; and therefore fome portion of the extractive matter of the liquids, and the whole of the folids, might have been added to the fyftem, and might, notwithftanding the difcharge by urine, feemingly, have anfwered all the purpofes of nutrition, and might, of courfe, have acted as a barrier to the increafing emaciation.

And, moreover, that the lofs of fubftance in Diabetes is not connected with any quality of the urine is farther fubftantiated, when it is confidered, that allowing fome fmall increafed difcharge of urinary extractive matter in thefe cafes beyond what takes place in health, it is well known that fuch patients are

ſaved any loſs from the fluids or ſolids of the body by the abſence of the wonted excretion of the ſurface, perſpiration ſenſible and inſenſible being as it were annihilated from the torpor and total inaction of the perſpiratory veſſels. And hence it would appear, by the defect of perſpiration, a quantity of fluid and its extract is ſaved, and retained in the ſyſtem, and perhaps more than a counter-balance to any increaſed quantity of extractive matter diſcharged by urine: And by this train of thinking, I have been induced to conclude, that the quality of the urine cannot be conſidered as the cauſe of the ſhrinking in Diabetes.

