# To Dr. Batty

**Published:** 1801-02

**Authors:** R. Lubbock

**Affiliations:** Norwich


### *To Dr. BATTY.*

*My dear Sir,*

*YOU will excuse my troubling you with this note, concerning an Error which has been committed in the printing off the sheets of my Communications on Diabetes, in the last Number of the Medical Journal. I find that the pages have not been printed off in the order in which they were written, so that in the perusal a great want of method and arrangement must necessarily arise. This being the case, I have to ask it as a favor of you and your Colleagues, that you would print in a conspicuous part of the next Number of your Journal, (and not among the Errata) an address to your Readers, requesting them, in looking over my Remarks, to insert the paragraph, at the foot of the 66th page, beginning with the words,* "And in treating of the extractive matter," *together with all the following paragraphs immediately before the paragraph in page 62, beginning with the words,* "But in ſpeaking upon this ſubject;" *by which insertion the order will be restored, and the Error corrected. I am, &c.*

